# The Prognostic Value of Programmed Death-Ligand 1 in a Chinese Cohort With Clear Cell Renal Cell Carcinoma

**DOI:** 10.3389/fonc.2019.00879

**Published:** 2019-11-25

**Authors:** Wen-jun Xiao, Fu-jiang Xu, Xuan Zhang, Shu-xian Zhou, Hai-liang Zhang, Bo Dai, Yao Zhu, Guo-hai Shi, Yi-jun Shen, Yi-ping Zhu, Yuan-yuan Qu, Jian-yuan Zhao, Ding-wei Ye

**Affiliations:** ^1^Department of Urology, Fudan University Shanghai Cancer Center, Institutes of Biomedical Sciences, and School of Life Sciences, Fudan University, Shanghai, China; ^2^Department of Oncology, Shanghai Medical College, Fudan University, Shanghai, China; ^3^Collaborative Innovation Center for Genetics and Development, Fudan University, Shanghai, China

**Keywords:** programmed death–ligand 1, renal cell carcinoma, immunohistochemistry, real-time polymerase chain reaction, overall survival, progression-free survival

## Abstract

**Objective:** To investigate the association between tumor PD-L1 expression and patient survival to determine whether PD-L1 represents an independent prognostic feature for patients with non-metastatic clear cell renal cell carcinoma (RCC).

**Patients and Methods:** The tissue bank of the Fudan University Shanghai Cancer Center was queried to identity tissue samples of patients treated with radical nephrectomy, for non-metastatic sporadic clear cell RCC (ccRCC) between 2008 and 2015. Real-time polymerase chain reaction and immunohistochemistry staining was performed to detect the expression level of PD-L1 in paired cancer tissue and paracancerous tissue.

**Results:** Three-hundred-and-thirty patients were enrolled in this study, with a mean age of 55.0 years at surgery and a mean tumor size of 5.2 cm. Two-hundred-and-forty-two (73.3%) and 88 (26.7%) patients showed a high and low expression of PD-L1 mRNA, respectively, while 254 patients had positive PD-L1 immunohistochemistry staining. Two-hundred-and-ninety-two patients had consistent results for mRNA and the PD-L1 protein based on these different detection methods. Patients with high PD-L1 expression were more likely to exhibit adverse pathologic features including an advanced T stage (*P* = 0.002) and lymph node metastasis (*P* = 0.044). The Kaplan–Meier curves of PFS and OS stratified by PD-L1 expression had a statistically significant difference. PD-L1 expression maintained a significant predictive role for PFS and OS in the multivariate cox model.

**Conclusions:** Our data suggests that PD-L1 correlates with prognosis in RCC and targeting the PD-1/PD-L1 pathway should be considered in the treatment of RCC patients.

## Introduction

Immunotherapy plays an important role in the treatment of renal cell carcinoma (RCC) (1, 2). Cytokines have been used for a long time as the mainstay of immunotherapy for clear cell RCC (ccRCC). However, due to the limited effectiveness of cytokines, it is urgent to investigate novel modalities with different mechanisms for treating tumors. Immune checkpoint therapy is one such method that enhances the antitumor immune response by regulating the activity of T cells through a combination of costimulatory signals. Under the physiological condition, these immune checkpoints can regulate the immune response to a certain strength and breadth, so as to avoid damage to normal tissue. In the process of development or recurrence of cancer, immune checkpoints may become one of the main reasons of immune tolerance and could be considered as a therapeutic target (3).

Programmed death-1(PD-1) and its ligand programmed death-ligand 1 (PD-L1) have recently become research hotspots in immunotherapy. New drugs targeting the PD-1/PD-L1 axis were reported to give very promising clinical responses in patients with various types of cancer (4–6). Earlier studies have reported conflicting data on correlations between PD-L1 expression in different solid tumor types with both improved (7, 8) and poor prognosis (9, 10). As far as RCC is concerned, patients with PD-L1-positive tumors are at significant risk of cancer progression, cancer-specific death, and overall mortality (9, 11). Drugs such as Nivolamub alone or in conjunction with Ipilimumab and Avelumab, or pembrolizumab with axitinib, have been approved for use in patients with metastatic ccRCC (12, 13). However, there is still not enough evidence for the use of these drugs in non-metastatic diseases before or after nephrectomy. Here, we focus on non-metastatic patients and investigate the association between tumor PD-L1 expression and patient survival, to determine the prognostic value in Chinese patients.

## Materials and Methods

### Patients and Samples Collection

The tissue bank of Fudan University Shanghai Cancer Center (FUSCC) was queried to identity tissue samples of patients treated with radical nephrectomy for sporadic non-metastatic ccRCC, between 2008 and 2015. The institutional ethical committee has approved the informed consent provide for collecting the tissue into tissue bank. Tumor tissue samples paired with adjacent normal tissues were collected at the time of surgery and preserved under appropriate conditions only after informed consent was signed by individual patients. Clinical and pathological information were collected, including age at surgery, gender, clinical manifestation, laterality, tumor size, and the International Society for Urologic Pathology (ISUP) grade and tumor-node-metastasis (TNM) stage groupings (14, 15). Clinical stages were determined according to the seventh edition of the American Joint Committee on Cancer (AJCC) staging manual (16). Cases with unavailable clinical and pathological characteristics were excluded.

### Quantitative PCR Analysis

Total RNA was isolated using the TRIzol reagent (Invitrogen, Carlsbad, CA), followed by reverse-transcribed reaction using the SuperScript First Strand cDNA system (Invitrogen, Carlsbad, CA). Real-time PCR was performed using the ABI Prism 7900 sequence detection system (Applied Biosystems) with β-actin as an internal reference. The forward and reverse primers for *PD-L1* were 5′-TGGCATTTGCTGAACGCATTT-3′ and 5′-TGCAGCCAGGTCTAATTGTTTT-3′. For the β-actin gene, the sense and antisense primers were 5′-GCCGACAGGATGCAGAAGGAGATCA-3′ and 5′-AAGCATTTGCGGTGGACGATGGA-3′, respectively. For each assay, a total of 10 μl reaction mixture was prepared using SYBR Green PCR master mix (Applied Biosystems) according to the manufacturer's instructions. Specific cycling conditions for β-action and *PD-L1* were carried out as follows: denaturation at 95°C for 3 min, followed by 45 cycles of denaturation at 95°C for 20 s, annealing at 60°C for 20 s, extension at 68°C for 20 s, and measurement at 80°C for 20 s, followed by a final extension at 72°C for 5 min. To confirm the specificity of amplification, melting curve analyses were performed and PCR products were sequenced and resolved in a 1% agarose gel. The *PD-L1* mRNA expression was represented as ΔCt = Ct(PD-L1) – ΔCt(β-actin), and the relative expression of PD-*L1* in ccRCC was measured using the ratio of *PD-L1* expression in ccRCC/matched normal tissues. “Low *PD-L1* expression” denotes the ratio of *PD-L1* mRNA expression in ccRCC/matched normal tissues of <1.5; “high *PD-L1* expression” denotes the ratio of *PD-L1* mRNA expression in ccRCC/matched normal tissues of >1.5.

### Immunohistochemistry Staining

PD-L1 immunostaining of tissue samples was performed using a rabbit monoclonal anti-PD-L1 antibody (1:150 dilution; Cell Signaling Technology, Billerica, MA, USA) and the Envision detection kit (Dako, Carpinteria, CA, USA). Tissue sections (4 μm thick) obtained from archived formalin-fixed, paraffin-embedded tissue blocks were deparaffinized in a xylene series and rehydrated with a graded ethanol series. Thereafter, endogenous peroxidase was quenched by incubation in 0.3% H_2_O_2_ for 15 min at 37°C, and non-specific binding was blocked by incubation in 10% normal goat serum for 60 min at room temperature. For antigen retrieval, sections were autoclaved in 0.01 M sodium citrate buffer, pH 6.0 (at 20 psi) for 10 min. Tissue sections were then incubated overnight with primary antibody at 4°C. Chromogenic antigen detection was carried out using a peroxidase-conjugated secondary antibody (60 min incubation) and DAB reagents (1 min incubation; Envision detection kit, Dako, Carpinteria, CA, USA). Tissue sections were counterstained with Meyer's hematoxylin (Thermo Fisher Scientific, Waltham, MA, USA). All sections were examined and scored by two experienced pathologists blinded to all clinical data in an open discussion. The percentages of tumor cells with positive PD-L1 staining were quantified. In accordance with previous studies, PD-L1 tumor positivity was defined as membrane staining of ≥5% tumor cells (17, 18).

### Statistical Analysis

The associations of *PD-L1* mRNA expression or IHC expression with clinical and pathological features were evaluated using the Student's *t*-test for continuous variables and the χ^2^-test for categorical variables. Progression-free survival (PFS) and over survival (OS) was measured as the time from the date of surgery to the corresponding endpoints or until the most recent follow-up visit for censored (living) patients. Kaplan–Meier method was used for estimating PFS and OS, and a Log-rank test was taken to compare different Kaplan–Meier curves. Clinical outcomes were evaluated using Cox proportional hazards regression models univariately and multivariately, between groups with different levels of *PD-L1* mRNA or IHC expression. The level of significance was defined as *p* < 0.05. SPSS software V13.0 (SPSS, Chicago, IL) was used for all statistical analyses.

## Results

### Patient Characteristics and Follow-Up

In total, 330 patients were enrolled in this study, with a mean age of 55.0 years at surgery and a mean tumor size of 5.2 cm. Males (66.4%) were approximately twice as many as females (33.6%). The demographics and disease characteristics were summarized in Table 1. At last follow-up, 98 of the 330 patients had died, with a median follow-up time of 47 (range 7–100) months. Among the 232 remaining patients, the median duration of follow-up was 69 (range 35–110) months. Survival rates at 1, 3, and 5 years following nephrectomy were 99.4, 90.6, and 77.3%, respectively.

**Table 1:** Clinicopathological characteristics and survival information stratified by PD-L1 expression status.

**Variable**	**Entire group (*n* = 330)**	**PD-L1 expression with consistent results for IHC and RT-PCR (*****n*** **=** **292)**			**PD-L1 mRNA expression (*****n*** **=** **330)**			**PD-L1 IHC expression (*****n*** **=** **330)**		
		**Low mRNA and negative IHC (*n* = 63)**	**high mRNA and positive IHC (*n* = 229)**	***P* value**	**Low (*n* = 88)**	**High (*n* = 242)**	***P* value**	**Negative (*n* = 76)**	**Positive (*n* = 254)**	***P* value**
Age at surgery (y, mean±SD)	55.0 ± 11.9	53.8 ± 10.3	55.5 ± 12.4	0.324	53.9 ± 10.4	55.4 ± 12.4	0.289	53.9 ± 10.4	55.3 ± 12.3	0.338
Sex (*n*, %)				0.531			0.370			0.691
Male	219 (66.4)	40 (63.5)	155 (67.7)		55 (62.5)	164 (67.8)		49 (64.5)	170 (66.9)	
Female	111 (33.6)	23 (36.5)	74 (32.3)		33 (37.5)	78 (32.2)		27 (35.5)	84 (33.1)	
Clinical manifestation (*n*, %)				0.353			0.333			0.460
Incidental	220 (66.7)	39 (61.9)	156 (68.1)		55 (62.5)	165 (68.2)		48 (63.2)	172 (67.7)	
Symptomatic	110 (33.3)	24 (38.1)	73 (31.9)		33 (37.5)	77 (31.8)		28 (36.8)	82 (32.3)	
Laterality (*n*, %)				0.698			0.573			0.560
Left	164 (49.7)	32 (50.8)	110 (48.0)		46 (52.3)	118 (48.8)		40 (52.6)	124 (48.8)	
Right	166 (50.3)	31 (49.2)	119 (52.0)		42 (47.7)	124 (51.2)		36 (47.4)	130 (51.2)	
Tumor size (cm, mean ± SD)	5.2 ± 2.4	5.2 ± 2.7	5.1 ± 2.3	0.770	5.3 ± 2.6	5.1 ± 2.3	0.701	5.3 ± 2.7	5.1 ± 2.3	0.491
T stage at presentation (n, %)				**0.002**			** <0.001**			**0.032**
T1-T2	295 (89.4)	63 (100.0)	197 (86.0)		88 (100.0)	207 (85.5)		73 (96.1)	222 (87.4)	
T3-T4	35 (10.6)	0 (0.0)	32 (14.0)		0 (0.0)	35 (14.5)		3 (3.9)	32 (12.6)	
N stage at presentation (n, %)				**0.044**			**0.021**			**0.036**
N0	316 (95.8)	63 (100.0)	215 (93.9)		88 (100.0)	228 (94.2)		76 (100.0)	240 (94.5	
N1	14 (4.2)	0 (0.0)	14 (6.1)		0 (0.0)	14 (5.8)		0 (0.0)	14 (5.5)	
ISUP grade (n, %)				0.172			**0.010**			0.142
1–2	171 (51.8)	35 (55.6)	105 (45.9)		56 (63.6)	115 (47.5)		45 (59.2)	126 (49.6)	
3–4	159 (48.2)	28 (44.4)	124 (54.1)		32 (36.4)	127 (52.5)		31 (40.8)	128 (50.4)	
Progression-free survival (months, median)	74	NA	50	**<0.001**	NA	50	**<0.001**	86	56	**<0.001**
Overall survival				**<0.001**			**<0.001**			**0.001**
(months, median)	100	NA	100		NA	98		NA	100	

### PD-L1 Expression and Its Relation to Patient Baseline Features

**Figure 1:**
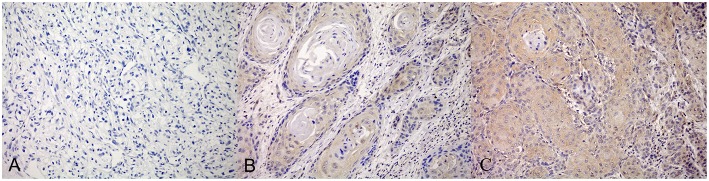
PD-L1 expression detected by immunohistochemistry (IHC) staining. The negetive **(A)**, weakly positive **(B)** and strongly positive **(C)** were shown separately.

Among 330 ccRCC patients, 242 (73.3%) and 88 (26.7%) showed a high and low expression of *PD-L1* mRNA, respectively, while 254 patients showed positive PD-L1 IHC staining. Different methods might have controversial results for detection of PD-L1. As shown in Figure 1, most cases with high or medium *PD-L1* mRNA expression had positive PD-L1 IHC expression, however, there were still some cases with negative PD-L1 IHC expression. On the other hand, most cases with low *PD-L1* mRNA expression had negative PD-L1 IHC expression, while a small percentage of cases had positive PD-L1 IHC expression. The subgroup of 292 cases was then established which had consistent results for PD-L1 mRNA and IHC expression.

The comparison of the clinical and pathologic features correlating with *PD-L1*mRNA or IHC expression is also shown in Table 1. Patients with high *PD-L1*mRNA expression were more likely to exhibit advanced pathologic features including poorer tumor grade (*P* = 0.010), advanced T stage (*P* = 0.034), and lymph node metastasis (*P* = 0.005). There was no statistically significant difference in age and sex among patients with different *PD-L1* mRNA expression (*P* = 0.858, 0.109, respectively). Patients with positive PD-L1 IHC expression had almost the same clinical and pathologic features as patients with high *PD-L1* mRNA expression, except for tumor size.

### PD-L1 Expression and Survival Analysis

**Figure 2:**
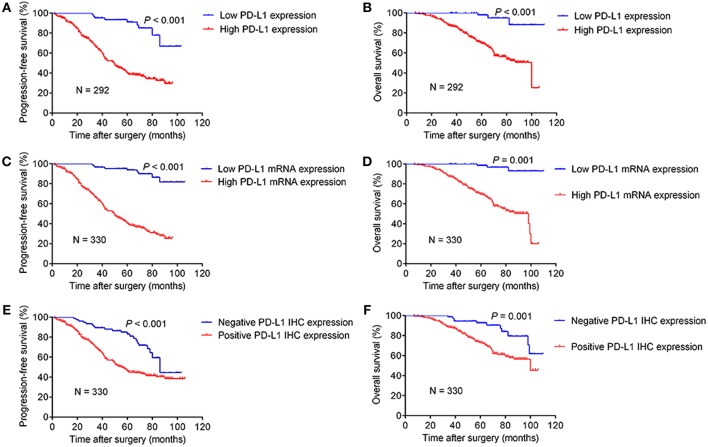
Kaplan–Meier curves of progression-free survival and overall survival stratified by PD-L1 expression level. The subgroups shown in **(A,B)** were 292 cases with consistent results for PD-L1 mRNA and IHC expression, while the remainder of the figure **(C–F)** refered to the whole cohort of 330 cases.

The Kaplan–Meier curves of PFS and OS stratified by PD-L1 expression are shown in Figure 2. In the subgroup of 292 cases with consistent results for PD-L1 mRNA and IHC expression, the median PFS and OS for patients with a high expression of PD-L1 was 50 months (95% CI: 42–58) and 100 months (95% CI: 74–), respectively. The PFS and OS stratified by PD-L1 expression had a statistically significant difference (*P* < 0.001). The median PFS and OS for patients with low *PD-L1* mRNA expression was longer than the subgroups with high *PD-L1* mRNA expression (Figures 2C,D), while the subgroup with negative PD-L1 IHC staining had better PFS and OS compared to the subgroup with positive staining (Figures 2E,F).

### Prognostic Factors Analysis

The results of univariate and multivariate Cox regression analyses for PFS were described in Table 2. The univariate analyses revealed that older age at surgery, advanced T stage, lymph node metastasis, poor tumor grade, positive PD-L1 IHC staining, and high *PD-L1* mRNA expression may be associated with lower PFS. In the multivariate analysis, most of these factors remained statistically significant except for the age at surgery. Table 3 summarizes the results of the univariate and multivariate cox regression analysis for OS. The univariate analysis revealed that prognostic factors of OS were the same as those of PFS, including older age at surgery, advanced T stage, lymph node metastasis, poor tumor grade, positive PD-L1 IHC staining, and high *PD-L1* mRNA expression. Multivariate analysis revealed that most of these factors maintained a significant predictive role for shorter OS. Notably, our study indicates that the expression stratification of PD-L1 based upon qRT-PCT or IHC staining may yield a different prognostic value for OS, that is, high PD-L1 mRNA expression was an independent prognostic factor for OS, however, positive PD-L1 IHC expression nearly lost significance after adjusting for covariants.

**Table 2:** Univariate and multivariate Cox regression analyses of PFS.

**Covariates**	**Univariate analysis^a^**		**Multivariate analysis^b^**		**Multivariate analysis^c^**		**Multivariate analysis^d^**	
	**HR (95%CI)**	***P*-value**	**HR (95%CI)**	***P*-value**	**HR (95%CI)**	***P*-value**	**HR (95%CI)**	***P*-value**
Age at surgery	**1.014 (1.001–1.028)**	**0.041**	1.005 (0.991–1.019)	0.486	1.004 (0.991–1.017)	0.566	1.003 (0.990–1.017)	0.623
Sex (male vs. female)	0.827 (0.588–1.163)	0.274						
Clinical manifestation (incidental vs. symptomatic)	1.051 (0.758–1.458)	0.764						
Laterality (left vs. right)	1.053 (0.771–1.437)	0.746						
Tumor size	1.036 (0.976–1.099)	0.244						
T stage (T1-T2 vs. T3-T4)	**8.107 (5.350–12.284)**	**<0.001**	**3.511 (2.196–5.611)**	**<0.001**	**3.802 (2.420–5.973)**	**<0.001**	**4.507 (2.844–7.142)**	**<0.001**
N stage (N0 vs. N1)	**11.108 (6.151–20.062)**	**<0.001**	**4.083 (2.179–7.650)**	**<0.001**	**3.861 (2.076–7.181)**	**<0.001**	**3.945 (2.106–7.389)**	**<0.001**
ISUP grade (1-2 vs. 3-4)	**2.628 (1.894–3.647)**	**<0.001**	**2.123 (1.482–3.043)**	**<0.001**	**2.056 (1.463–2.890)**	**<0.001**	**2.170 (1.542–3.054)**	**<0.001**
PD-L1 mRNA expression (low vs. high)	**9.693 (4.936–19.032)**	**<0.001**	**5.305 (2.679–10.502)**	**<0.001**	**7.940 (4.017–15.694)**	**<0.001**		
PD-L1 IHC expression (negative vs. positive)	**2.444 (1.557–3.836)**	**<0.001**	**5.305 (2.679–10.502)**	**<0.001**			**2.043 (1.289–3.238)**	**0.002**

a *Univariate analysis was performed in the entire cohort (n = 330)*.

b *Multivariate cox model including PD-L1 expression and covariates in the subgroup that patients with low PD-L1 mRNA expression and negative PD-L1 IHC expression or with high PD-L1 mRNA expression and positive PD-L1 IHC expression (n = 292)*.

c *Multivariate cox model including PD-L1 mRNA expression and covariates in the entire cohort (n = 330)*.

d *Multivariate cox model including PD-L1 IHC expression and covariates in the entire cohort (n = 330)*.

**Table 3:** Univariate and multivariate Cox regression analyses of OS.

	**Univariate analysis^a^**		**Multivariate analysis^b^**		**Multivariate analysis^c^**		**Multivariate analysis^d^**	
**Covariates**	**HR (95%CI)**	***P*-value**	**HR (95%CI)**	***P*-value**	**HR (95%CI)**	***P*-value**	**HR (95%CI)**	***P*-value**
Age at surgery	**1.017 (1.001–1.034)**	**0.041**	1.008 (0.991–1.025)	0.352	1.008 (0.992–1.024)	0.328	1.009 (0.992–1.025)	0.303
Sex (male vs. female)	1.073 (0.705–1.631)	0.743						
Clinical manifestation (incidental vs. symptomatic)	1.007 (0.660–1.538)	0.973						
Laterality (left vs. right)	1.025 (0.690–1.524)	0.902						
Tumor size	0.981 (0.902–1.068)	0.661						
T stage (T1-T2 vs. T3-T4)	**9.857 (6.324–15.365)**	**<0.001**	**3.977 (2.370–6.673)**	**<0.001**	**4.499 (2.734–7.405)**	**<0.001**	**5.392 (3.237–8.981)**	**<0.001**
N stage (N0 vs. N1)	**12.496 (6.644–23.503)**	**<0.001**	**4.194 (2.143–8.208)**	**<0.001**	**3.857 (1.996–7.452)**	**<0.001**	**3.898 (2.006–7.575)**	**<0.001**
ISUP grade (2 vs. 3 or 4)	**2.853 (1.868–4.358)**	**<0.001**	**1.943 (1.326–3.414)**	**0.009**	**1.783 (1.134–2.802)**	**0.012**	**1.896 (1.202–2.991)**	**0.006**
PD-L1 mRNA expression (low vs. high)	**16.162 (5.093–51.290)**	**<0.001**	**2.009 (1.237–3.263)**	**0.005**	**11.067 (3.443–35.579)**	**<0.001**		
PD-L1 IHC expression (negative vs. positive)	**2.895 (1.537–5.451)**	**<0.001**	**2.009 (1.237–3.263)**	**0.005**			**1.970 (1.025–3.787)**	**0.042**

a *Univariate analysis was performed in the entire cohort (n = 330)*.

b *Multivariate cox model including PD-L1 expression and covariates in the subgroup that patients with low PD-L1 mRNA expression and negative PD-L1 IHC expression or with high PD-L1 mRNA expression and positive PD-L1 IHC expression (n = 292)*.

c *Multivariate cox model including PD-L1 mRNA expression and covariates in the entire cohort (n = 330)*.

d *Multivariate cox model including PD-L1 IHC expression and covariates in the entire cohort (n = 330)*.

## Discussion

PD-L1 is a cell surface glycoprotein, which was found in 1999 and belonged to the B7 family of T cell costimulatory molecules (19). Typically, PD-L1 is constitutively expressed in macrophages (19). However, PD-L1 could also express in tumor cells, which may be involved in the tumor immune escape: inhibiting an immune response mediated by tumor-antigen-specific T cells, inducing apoptosis in T cells, reducing the secretion of cytokines, and activating the cytotoxic T cells (20, 21). Over the last 20 years, the field of immunology has achieved a better understanding of the mechanisms underlying the activation of the host immune cell and the costimulation of T-cells (22). In mice, a high expression of PD-L1 can reverse the anti-tumor effect of tumor-antigen-specific T cells, while blocking PD-L1 could promote the anti-tumor immune response of T cells (23). PD-L1 may be the only costimulatory molecules of T cells that are associated with the poor prognosis in malignant tumors. Therefore, PD-L1 could be an important blockade target used to enhance the anti-tumor immune response (24).

RCC is an immunogenic tumor associated with high levels of infiltrating immune cells, mainly mononuclear cells. These mononuclear cells are predominantly composed of T cells. As previously reported, PD-L1 expressed in tumor cells of RCC, influencing the biological behavior and correlating independently with poor cancer-specific survival rates, while PD-1 expressed in infiltrating mononuclear immune cells within RCC (9, 11). Most published studies used IHC to evaluate PD-L1 expression (9, 11, 25). However, IHC-based detection of PD-L1 has some technical issues that affect the accuracy of PD-L1 IHC staining. On the one hand, since there are various PD-L1 antibodies that can be obtained commercially, diverse antibodies used in different studies may have different sensitivity (13). On the other hand, the IHC staining procedures and scoring methods are difficult to standardize. The optimal cut-off value for PD-L1 expression is also difficult to establish. The section evaluation depends on pathologists, so it may be semi-quantitative and subjective. Until now, there have been few studies on the expression and prognostic value of PD-L1 in RCC with a large cohort of Chinese patients.

This study included 330 cases of non-metastatic Chinese ccRCC patients and explored both qRT-PCR, which is highly sensitive and objective, and IHC to detect the PD-L1 expression in paired tumor tissues and adjacent normal tissues. Our results revealed that although high PD-L1 mRNA expression was an independent prognostic factor for OS, positive PD-L1 IHC expression nearly lost significance after adjusting for covariants, which indicated that IHC staining alone was not accurate enough to define the PD-L1expression level. To overcome the challenge of normalizing the expression levels, we used absolute values of cancerous tissue normalized to the values of corresponding paracancerous tissue to establish a measurable method of PD-L1 expression. With this approach we defined a 2-tier system for expression evaluation. The results of qRT-PCR in the present study should be more quantitative and reliable. It may be more persuasive to provide results based on two detection methods used together.

Our results showed that the high expression of PD-L1 in tumor cells correlated to poorer pathological features, including a greater tumor size, high nuclear grade, lymph node metastasis, and more advanced stages. In addition, patients with high PD-L1 expression had a significantly increased risk of disease-specific mortality and overall mortality. Our work provides further evidence in the Chinese population that the functional level of the PD-L1/PD-1 pathway weakens the body's immune surveillance, thereby contributing to tumor progression.

However, the present study also has some limitations. First, the expression of PD-L1 is not uniform even in the primary tumor. Near the margin of the tumor, the PD-L1 expression was induced by external factors produced by juxtaposed T lymphocytes. The PD-L1 expression of other parts is more likely to be affected by intrinsic factors and the molecular mechanism. The expression pattern of PD-L1 in different tumor locations could not be distinguished by qRT-PCR in the present study. Second, due to the heterogeneity of the tumor, the expressions of PD-L1 in primary and metastatic tumor cells were not the same. In some cases, the expression of PD-L1 in the primary tumor is negative, while it is positive in the metastatic tumor (25, 26). That is why anti-PD-L1 therapy was still effective, even if PD-L1 was negative in the primary tumor (25, 27). But in the present study the metastatic tumor was not available, so, we could not compare the expression of PD-L1 between the primary tumor and metastatic tumor. Third, the postoperative medical treatment was not included in this study, which may cause some bias.

## Conclusions

Our data thus suggests that PD-L1 correlated with the prognosis in ccRCC and targeting the PD-1/PD-L1 pathway may be therapeutically efficacious and should thus be considered in the treatment of RCC patients.

## Data Availability Statement

The raw data supporting the conclusions of this manuscript will be made available by the authors, without undue reservation, to any qualified researcher.

## Ethics Statement

This study was carried out in accordance with the recommendations of the local ethics committee with written informed consent from all subjects. All subjects gave written informed consent in accordance with the Declaration of Helsinki. The protocol was approved by the Fudan University Shanghai Cancer Center ethics committee.

## Author Contributions

WX, YQ, and JZ conceived the idea. YQ, HZ, GS, BD, YZ, and YS provided patient data and material. YQ, FX, YPZ, and XZ contributed to the sample-preparations. FX, XZ, and SZ carried out the laboratory analyses. WX and YQ performed the statistics. WX, FX, and JZ analyzed and interpreted the data. YQ, JZ, and DY were involved in the planning and supervising. WX, FX, and YQ drafted the manuscript and designed the figures. WX, FX, YQ, and DY wrote the manuscript, with contributions from the other authors. All authors read and approved the final manuscript.

### Conflict of Interest

The authors declare that the research was conducted in the absence of any commercial or financial relationships that could be construed as a potential conflict of interest.
